# Supplementary material for: Spherical arena reveals optokinetic response tuning to stimulus location, size, and frequency across entire visual field of larval zebrafish
Source: eLife. 2021 Jun 8;10:e63355. doi: 10.7554/eLife.63355 (PMC8233042; doi:10.7554/eLife.63355)
Supplement: Supplementary file 1. — (A) The arena cross-section, (B) stimulus parameters for position dependence experiments, (C) absolute positions of fish and setup elements in different experiments, (D) stimulus parameters for control experiments, (E) parameters for whole-field and hemisphere stimuli, (F) stimulus parameters for frequency dependence experiments, and (G) stimulus parameters for size dependence experiments. [file elife-63355-supp1.docx]

## Spherical arena reveals optokinetic response tuning to stimulus location, size, and frequency across entire visual field of larval zebrafish

*Supplementary Tables*

## Authors

Florian A. Dehmelt^1^*, Rebecca Meier^1^*, Julian Hinz^1^*^#^, Takeshi Yoshimatsu^2^,
Clara A. Simacek^1^, Ruoyu Huang^1^, Kun Wang^1^, Tom Baden^2^, Aristides B. Arrenberg^1¶^

** These authors contributed equally to this work.*

## Affiliations

1: University of Tübingen, Werner Reichardt Centre for Integrative Neuroscience and Institute of Neurobiology, 72076 Tübingen, Germany

2: Sussex Neuroscience, School of Life Sciences, University of Sussex, UK

^#^ Current address: Friedrich Miescher Institute for Biomedical Research (FMI), Basel, Switzerland

## Correspondence

^¶^Correspondence should be addressed to A.B.A.
(e-mail: aristides.arrenberg@uni-tuebingen.de)

## Supplementary Tables

**Supplementary File 1A. Arena cross-section.** Elevation of LED tile centres, from top ribbon (ribbon number +5, near the north pole of the sphere) to bottom ribbon (ribbon number -5, near the South Pole). See **Figure 1–figure supplement 1a** for a graphical illustration. *As the left and right hemispheres of the arena are mirror-symmetric to one another, each ribbon contains the same number of tiles within each of the two hemispheres. **Because the structural scaffold is reinforced near the bottom of the sphere, ribbon -4 contains one fewer LED tile than ribbon +4. Other than that, the arrangement of LED tiles is almost mirror-symmetric from top to bottom as well.

| ribbon no. k | tiles per ribbon | elevation $\boldsymbol{\beta}_{\boldsymbol{k}}$of tile centre (degrees) |
| --- | --- | --- |
| +5 | 2* x 5 | 62.4 |
| +4 | 2 x 9** | 49.9 |
| +3 | 2 x 11 | 37.6 |
| +2 | 2 x 13 | 25.0 |
| +1 | 2 x 14 | 12.5 |
| 0 (equator) | 2 x 15 | 0 |
| -1 | 2 x 14 | -12.5 |
| -2 | 2 x 13 | -25.0 |
| -3 | 2 x 11 | -37.6 |
| -4 | 2 x 8** | -49.9 |
| -5 | 2 x 5 | -62.4 |

**Supplementary File 1B.** **Stimulus parameters (position dependence).** These stimuli consisted of a horizontally moving grating, cropped with a disk-shaped stimulus mask, and presented in one of 38 different locations across the visual field. Results shown in **Figure 3** and **Figure 4e-f**. The stimulus mask is determined by the azimuth $\alpha$ (degrees) and elevation $\beta$ (degrees) of its centre, as well as its size, given by the angle $\delta$ (degrees) it spans. The moving grating is characterised by its spatial frequency SF (cycles/degree), temporal frequency TF (cycles/sec), peak velocity v (deg/sec), and oscillation period T (sec).

| type | $\boldsymbol{\alpha}$ | $\boldsymbol{\beta}$ | $\boldsymbol{\delta}$ | SF | TF | v | T |
| --- | --- | --- | --- | --- | --- | --- | --- |
| D1 | -16 | 25 | 64 | 0.0611 | 0.7639 | 12.5 | 10 |
| D2 | -35 | 53 | 64 | 0.0611 | 0.7639 | 12.5 | 10 |
| D3 | -90 | 74 | 64 | 0.0611 | 0.7639 | 12.5 | 10 |
| D4 | -145 | 53 | 64 | 0.0611 | 0.7639 | 12.5 | 10 |
| D5 | -164 | 25 | 64 | 0.0611 | 0.7639 | 12.5 | 10 |
| D6 | -60 | 19 | 64 | 0.0611 | 0.7639 | 12.5 | 10 |
| D7 | -90 | 39 | 64 | 0.0611 | 0.7639 | 12.5 | 10 |
| D8 | -120 | 19 | 64 | 0.0611 | 0.7639 | 12.5 | 10 |
| D9 | -30 | 0 | 64 | 0.0611 | 0.7639 | 12.5 | 10 |
| D10 | -90 | 0 | 64 | 0.0611 | 0.7639 | 12.5 | 10 |
| D11 | -150 | 0 | 64 | 0.0611 | 0.7639 | 12.5 | 10 |
| D12 | -60 | -19 | 64 | 0.0611 | 0.7639 | 12.5 | 10 |
| D13 | -90 | -39 | 64 | 0.0611 | 0.7639 | 12.5 | 10 |
| D14 | -120 | -19 | 64 | 0.0611 | 0.7639 | 12.5 | 10 |
| D15 | -16 | -25 | 64 | 0.0611 | 0.7639 | 12.5 | 10 |
| D16 | -35 | -53 | 64 | 0.0611 | 0.7639 | 12.5 | 10 |
| D17 | -90 | -74 | 64 | 0.0611 | 0.7639 | 12.5 | 10 |
| D18 | -145 | -53 | 64 | 0.0611 | 0.7639 | 12.5 | 10 |
| D19 | -164 | -25 | 64 | 0.0611 | 0.7639 | 12.5 | 10 |
| D20-D38 | same as D1-D19, but with positive azimuth $\alpha$ (right hemisphere of arena) | | | | | | |

**Supplementary File 1C. Absolute positions of fish and setup elements in different experiments.** All positions and directions are given in environmental coordinates, i.e. the approximate cardinal directions (North etc.) of the laboratory, as well as Up and Down (away from or towards the core of the Earth). Most experiments, including control experiments, are of type 1 (**Figure 4a**). The only exceptions are the position-dependence experiments with rotated arena (type 2, **Figure 4b**), control experiments with upside-down embedding (type 3, **Figure 4c**), and control experiments with inverted IR illumination (type 4, **Figure 4d**). Three LED tiles in different parts of the visual field are included as examples. Positions are given in lab-centred geographic coordinates as (a,e), where a is the azimuth, e the elevation, and 0° azimuth is arbitrarily chosen to point South.

| experiments | type 1 | type 2 | type 3 | type 4 |  |
| --- | --- | --- | --- | --- | --- |
| infrared illumination at | Up (0°,+90°) | Up (0°,+90°) | Up (0°,+90°) | Down (0°,-90°) |  |
| camera at | Down (0°,-90°) | Down (0°,-90°) | Down (0°,-90°) | Up (0°,+90°) |  |
| flat face of glass triangle facing | Up (0°,+90°) | Up (0°,+90°) | Up (0°,+90°) | Up (0°,+90°) |  |
| dorsum facing | Up (0°,+90°) | Up (0°,+90°) | Down (0°,-90°) | Up (0°,+90°) |  |
| rostrum facing | South (0°,0°) | North (180°,0°) | South (0°,0°) | South (0°,0°) |  |
| arena axis A | South (0°,0°) | North (180°,0°) | South (0°,0°) | South (0°,0°) |  |
| arena axis B | West (90°,0°) | West (90°,0°) | West (90°,0°) | West (90°,0°) |  |
| arena axis C | Up (0°,+90°) | Down (0°,-90°) | Up (0°,+90°) | Up (0°,+90°) |  |
| LED tile #1 | Up, West, South (+35°,+53°) | Down, West, North (+145°,-53°) | Up, West, South (+35°,+53°) | Up, West, South (+35°,+53°) |  |
| LED tile #2 | East (-90°,0°) | East (-90°,0°) | East (-90°,0°) | East (-90°,0°) |  |
| LED tile #3 | Down, East, South (-16°,-25°) | Up, East, North (+164°,+25°) | Down, East, South (-16°,-25°) | Down, East, South (-16°,-25°) |  |

**Supplementary File 1D.** **Stimulus parameters (control experiments).** These stimuli consisted of a horizontally moving grating displayed on four flat, rectangular stimulus screens surrounding the larva. One pair of screens displayed stimuli visible to the left eye only, and the other pair displayed stimuli to the right eye only. Results shown in **Figure 3–figure supplement 5**.

| type | left eye stimulus | right eye stimulus | shown on panel |
| --- | --- | --- | --- |
| C0 | moving pattern | moving pattern | **a-c** |
| C1 | stationary pattern | moving pattern | **a-b** |
| C2 | moving pattern | stationary pattern | **a-b** |
| C3 | blank surface | moving pattern | **c-d, g-h** |
| C4 | moving pattern | blank surface | **c-d, g-h** |
| C5 | eye shielded | moving pattern | **e-f, g-h** |
| C6 | moving pattern | eye shielded | **e-f, g-h** |

**Supplementary File 1E.** **Stimulus parameters (whole-field and hemispheres).** These stimuli consisted of a horizontally moving grating, either covering the entire visual field or cropped to one of the 6 principal hemispheres (front, rear, upper, lower, left, right). Parameters and units as in **Supplementary File 1B.** Results shown in **Figure 3–figure supplement 4** and **Figure 4–figure supplement 1**.

| type | $\boldsymbol{\alpha}$ | $\boldsymbol{\beta}$ | $\boldsymbol{\delta}$ | SF | TF | v | T |
| --- | --- | --- | --- | --- | --- | --- | --- |
| H0 | 0 | 0 | 360 | 0.0611 | 0.7639 | 12.5 | 10 |
| H1 | 0 | 0 | 180 | 0.0611 | 0.7639 | 12.5 | 10 |
| H2 | 180 | 0 | 180 | 0.0611 | 0.7639 | 12.5 | 10 |
| H3 | 0 | 90 | 180 | 0.0611 | 0.7639 | 12.5 | 10 |
| H4 | 0 | -90 | 180 | 0.0611 | 0.7639 | 12.5 | 10 |
| H5 | 90 | 0 | 180 | 0.0611 | 0.7639 | 12.5 | 10 |
| H6 | -90 | 0 | 180 | 0.0611 | 0.7639 | 12.5 | 10 |

**Supplementary File 1F.** **Stimulus parameters (frequency dependence).** These stimuli consisted of a horizontally moving grating, cropped with a disk-shaped stimulus mask. At each location, 7 different spatial frequencies and thus velocities were used, while temporal frequency was held constant. Parameters and units as in **Supplementary File 1B.** Results shown in **Figure 5**.

| type | $\boldsymbol{\alpha}$ | $\boldsymbol{\beta}$ | $\boldsymbol{\delta}$ | SF | TF | v | T |
| --- | --- | --- | --- | --- | --- | --- | --- |
| F1 | -70 | 15 | 40 | 0.0181 | 0.7639 | 42.188 | 10 |
| F2 | -70 | 15 | 40 | 0.0272 | 0.7639 | 28.125 | 10 |
| F3 | -70 | 15 | 40 | 0.0407 | 0.7639 | 18.750 | 10 |
| F4 | -70 | 15 | 40 | 0.0611 | 0.7639 | 12.500 | 10 |
| F5 | -70 | 15 | 40 | 0.0917 | 0.7639 | 8.3333 | 10 |
| F6 | -70 | 15 | 40 | 0.1375 | 0.7639 | 5.5556 | 10 |
| F7 | -70 | 15 | 40 | 0.2063 | 0.7639 | 3.7037 | 10 |
| F8-F14 | same as F1-F7, but with azimuth $\alpha=-110$ and elevation $\beta=-15$ | | | | | | |
| F15-F21 | same as F1-F7, but with azimuth $\alpha=-28$ and elevation $\beta=15$ | | | | | | |
| F22-F42 | same as F1-F21, but with positive azimuth $\alpha$ (right hemisphere) | | | | | | |

**Supplementary File 1G.** **Stimulus parameters (size dependence).** These stimuli consisted of a horizontally moving grating, cropped with a disk-shaped stimulus mask. At each location, disks with 7 different, logarithmically spaced areas were shown. Parameters and units as in **Supplementary File 1B.** Results shown in **Figure 5**.

| type | $\boldsymbol{\alpha}$ | $\boldsymbol{\beta}$ | $\boldsymbol{\delta}$ | SF | TF | v | T |
| --- | --- | --- | --- | --- | --- | --- | --- |
| A1 | -70 | 15 | 3.58 | 0.0611 | 0.7639 | 12.5 | 10 |
| A2 | -70 | 15 | 7.16 | 0.0611 | 0.7639 | 12.5 | 10 |
| A3 | -70 | 15 | 14.3 | 0.0611 | 0.7639 | 12.5 | 10 |
| A4 | -70 | 15 | 28.7 | 0.0611 | 0.7639 | 12.5 | 10 |
| A5 | -70 | 15 | 57.9 | 0.0611 | 0.7639 | 12.5 | 10 |
| A6 | -70 | 15 | 120 | 0.0611 | 0.7639 | 12.5 | 10 |
| A7 | -70 | 15 | 360 | 0.0611 | 0.7639 | 12.5 | 10 |
| A8-A14 | same as A1-A7, but with azimuth $\alpha=-110$ and elevation $\beta=-15$ | | | | | | |
| A15-A21 | same as A1-A7, but with azimuth $\alpha=-28$ and elevation $\beta=15$ | | | | | | |
| A22-A42 | same as A1-A21, but with positive azimuth $\alpha$ (right hemisphere) | | | | | | |
