# Supplementary material for: Spherical arena reveals optokinetic response tuning to stimulus location, size, and frequency across entire visual field of larval zebrafish
Source: eLife. 2021 Jun 8;10:e63355. doi: 10.7554/eLife.63355 (PMC8233042; doi:10.7554/eLife.63355)
Supplement: Transparent reporting form [file elife-63355-transrepform.docx]

***eLife’s* transparent reporting form**

We encourage authors to provide detailed information *within their submission* to facilitate the interpretation and replication of experiments. Authors can upload supporting documentation to indicate the use of appropriate reporting guidelines for health-related research (see [EQUATOR Network](http://www.equator-network.org/%20)), life science research (see the [BioSharing Information Resource](https://biosharing.org/)), or the [ARRIVE guidelines](http://www.plosbiology.org/article/info:doi/10.1371/journal.pbio.1000412) for reporting work involving animal research. Where applicable, authors should refer to any relevant reporting standards documents in this form.

If you have any questions, please consult our Journal Policies and/or contact us: [editorial@elifesciences.org](mailto:editorial@elifesciences.org).

**Sample-size estimation**

- You should state whether an appropriate sample size was computed when the study was being designed
- You should state the statistical method of sample size computation and any required assumptions
- If no explicit power analysis was used, you should describe how you decided what sample (replicate) size (number) to use

Please outline where this information can be found within the submission (e.g., sections or figure legends), or explain why this information doesn’t apply to your submission:

Sample size was based on previous observations and publications regarding the expected variability of optokinetic performance across individual fish. Trial-to-trial variability can of course be reduced by averaging across repetitions. But because the length of an experiment is limited by technical and biological factors, there is a trade-off between the number of different stimuli to show, and how often each one is repeated. The required dense sampling of the visual field thus allowed only two repetitions per trial and larva of each stimulus type, and we converged on a sample size between 7 and 10 animals for the different types of experiments.

**Replicates**

- You should report how often each experiment was performed
- You should include a definition of biological versus technical replication
- The data obtained should be provided and sufficient information should be provided to indicate the number of independent biological and/or technical replicates
- If you encountered any outliers, you should describe how these were handled
- Criteria for exclusion/inclusion of data should be clearly stated
- High-throughput sequence data should be uploaded before submission, with a private link for reviewers provided (these are available from both GEO and ArrayExpress)

Please outline where this information can be found within the submission (e.g., sections or figure legends), or explain why this information doesn’t apply to your submission:

All experiments were performed only once per larva. Technical replication is achieved through repeated presentation of stimuli and recording of behaviour. Figure 2, the Results section and Tables 1-3 explain how each oscillating stimulus was presented for extended periods of time, and each such presentation was repeated twice per stimulus type for each larva. Biological replication is achieved through sample size, i.e. by repeating the experiment for multiple larvae. Criteria for removing invalid trials are described in Figure 2, Figure 2-figure supplement 2, and the Results section (subsection “Stimulus position dependence of the optokinetic response”). Sample sizes are indicated in the figure legends of Figures 3 to 5.

**Statistical reporting**

- Statistical analysis methods should be described and justified
- Raw data should be presented in figures whenever informative to do so (typically when N per group is less than 10)
- For each experiment, you should identify the statistical tests used, exact values of N, definitions of center, methods of multiple test correction, and dispersion and precision measures (e.g., mean, median, SD, SEM, confidence intervals; and, for the major substantive results, a measure of effect size (e.g., Pearson's r, Cohen's d)
- Report exact p-values wherever possible alongside the summary statistics and 95% confidence intervals. These should be reported for all key questions and not only when the p-value is less than 0.05.

Please outline where this information can be found within the submission (e.g., sections or figure legends), or explain why this information doesn’t apply to your submission:

We performed a permutation test to assess whether stimuli above the equator elicit significantly stronger OKR gains than those below. This test is described in the Results section, subsection “Stimulus position dependence of the optokinetic response”. We provide the corresponding MATLAB code including all parameters as Supplementary Code 5.

(For large datasets, or papers with a very large number of statistical tests, you may upload a single table file with tests, Ns, etc., with reference to sections in the manuscript.)

**Group allocation**

- Indicate how samples were allocated into experimental groups (in the case of clinical studies, please specify allocation to treatment method); if randomization was used, please also state if restricted randomization was applied
- Indicate if masking was used during group allocation, data collection and/or data analysis

Please outline where this information can be found within the submission (e.g., sections or figure legends), or explain why this information doesn’t apply to your submission:

This information does not apply. Different groups were generally studied at different times. Because of the rapid development of larvae, fish were crossed just in time for the experiments, and the larvae issued from this crossing were used as part of the group currently studied. For Fig 3 - Fig Supp 5, all fish studied were part of all groups.

**Additional data files (“source data”)**

- We encourage you to upload relevant additional data files, such as numerical data that are represented as a graph in a figure, or as a summary table
- Where provided, these should be in the most useful format, and they can be uploaded as “Source data” files linked to a main figure or table
- Include model definition files including the full list of parameters used
- Include code used for data analysis (e.g., R, MatLab)
- Avoid stating that data files are “available upon request”

Please indicate the figures or tables for which source data files have been provided:

Processed data with corresponding MATLAB code is provided for Figures 3 to 6 as well as Figure 3-figure supplement 1, Figure 3-figure supplement 4, and Figure 6-figure supplement 1. Raw data examples are provided for Figures 3 to 5.
